# Preferences of adolescents – A dataset containing linked experimental task measures and register data

**DOI:** 10.1016/j.dib.2022.108088

**Published:** 2022-03-24

**Authors:** Dániel Horn, Hubert János Kiss, Tünde Lénárd

**Affiliations:** a Centre for Economic and Regional Studies, Institute of Economics, Tóth Kálmán utca 4., Budapest 1097, Hungary; b Department of Economics, Corvinus University of Budapest, Fővám tér 8., Budapest 1093, Hungary; c SOFI, Stockholm University, Stockholm SE-106 91, Sweden

**Keywords:** Adolescents, Altruism, Competitiveness, Cooperation, Risk preferences, Social preferences, Time preferences, Trust

## Abstract

Between March 2019 and March 2020, we visited 53 school groups (classes) in 9 Hungarian schools to measure time, risk, social and competitive preferences of 1108 secondary school students using incentivized laboratory experimental tasks.

We applied the unfolding brackets method to measure time preferences [1], and the bomb-risk elicitation task [2] to test risk preferences. For assessing competitive preferences, we utilized a real effort task (counting zeros) and used the three-round measure of competition [3]. We applied three different games to test social preferences: the dictator game, the trust game, and a simple public good game. We gave out vouchers for the school buffet to incentivize the experiments. We then took these anonymously measured preferences and connected them to the administrative panel of the National Assessment of Basic Competencies (NABC) using the hash codes provided by the Education Authority. We gain all additional information on gender, parental background, standardized test scores and school performance (grades) from the NABC data.

The dataset provides detailed insights into how preferences of upper secondary school students associate with educational outcomes, social background and gender. The database contains rich background data on the individual level. We observe students nested in classes (groups of students having most of their courses together) nested in schools which allows the analyst to see how the background variables relate to preferences not only on the individual level, but also within schools and within classes. Moreover, as we measure nine aspects of the four most widely used preferences at once, we can assess these relationships more precisely, conditional on correlated preferences. In an accompanying paper we study gender differences in preferences of adolescents using this dataset [4].

## Specifications Table

**Table:** 

Subject	Behavioral Economics, Experimental Economics
Specific subject area	Preferences of adolescents and their relationship to parental background, cognitive skills and school performance.
Type of data	Stata data file (.dta) Comma delimited file (.csv)
How data were acquired	Experimental data: gathered through a lab-in-the-field experimental series conducted in Hungarian classrooms Instrument: zTree 3.6.5 Administrative data: gathered from the database for National Assessment of Basic Competences, linked to the experimental data, anonymized and transformed
Data format	Raw data Filtered
Description of data collection	Experimental data were collected in Hungarian upper secondary schools in 53 experimental sessions. Schools were either suggested for us by educational providers or participated voluntarily. A session consisted of 8 computer-based tasks (using zTree [5]) that measured time, risk, social and competitive preferences of participants in an incentivized way. Standardized test scores and data on social background from previous years were linked to the experimental results.
Data source location	Institution: Institute of Economics, Centre for Economic and Regional Studies City/Town/Region: Budapest Country: Hungary
Data accessibility	Repository name: Mendeley Data Data identification number (permanent identifier, i.e. DOI number): 10.17632/96jt894stz.4 Direct link to the dataset: https://data.mendeley.com/datasets/96jt894stz/4
Related research article	D. Horn, H.J. Kiss, T. Lénárd, Gender differences in preferences of adolescents: Evidence from a large-scale classroom experiment, Journal of Economic Behavior & Organization. 194 (2022) 478–522. 10.1016/j.jebo.2021.12.015.

## Value of the Data

• The main strength of the dataset is that it contains a detailed map of preferences measured at once. Preferences might be correlated to each other, so measuring and analysing them without controlling for the other preference measures might lead to biased and inconclusive results. With this set of preferences, it is possible to pin down the effect of separate preferences by controlling for the effect of the other ones.
• This dataset is beneficial for any researcher wanting to explore how preferences relate to cognitive skills, school performance and how social background mediates this relationship.
• Since the dataset has school-class level data, it is possible to study the association of preferences and school performance on group level, i.e. if classes performing better academically have better aggregated social preferences.

## 1 Data Description

The file “Horn-Kiss-Lenard2021.dta” contains raw data from two sources linked together on individual level (the same data file in an open-source format is “Horn-Kiss-Lenard2021.csv”). One source was a series of experimental sessions where Hungarian upper secondary school students had to complete 8 computer-based tasks that were designed to assess their time, risk, social and competitive preferences. The other source was an administrative dataset of the National Assessment of Basic Competencies (NABC) containing test scores, school performance and social background data. The administrative variables are calculated from the 6th grade data of students, and we substituted missing values with 8th grade data, if the latter was available. All initial NABC variables were transformed so as to provide anonymity and be better suited for empirical analysis. The file “README.xlsx” provides a detailed description on each variable explaining how they were generated and if any filtering was applied on a variable.

The file “Horn-Kiss-Lenard2021.do” contains the Stata code used in [4] to analyze these data, and “Horn-Kiss-Lenard-eng.ztt” is the English translation of the zTree codes that were used in our experiment. All instructions that were read to participants before the experimental sessions can be found in the supplementary file “Instructions.pdf”, and “Figure1.png” shows the decision tree of the staircase method used to measure time preferences.

## 2 Experimental Design, Materials and Methods

### 2.1 Procedures

Our computer-based experiment was carried out in 53 classrooms across nine schools between March 2019 and March 2020^1^ in Hungary. Prior to beginning the project, we contacted all educational providers in the country that operated at least one secondary school (academic, vocational, or mixed) and obtained permission to perform the experimental sessions at their facilities. Providers who solely operate Special Vocational Schools were excluded. The schools in our sample were either recommended by the provider or actively expressed their intention to participate after receiving supporting feedback from the provider. Four of these schools are located in Budapest, and the other five in smaller rural municipalities across Hungary. Naturally, our selection of schools is not representative of Hungary's entire school population, since we mostly visited classes^2^ in academic programs and a few vocational secondary schools that offer maturity exams.

Participation in our experimental sessions was entirely voluntary and anonymous, which we informed students and their families about. We issued our data protection statement to all parents and children prior to the assessment, informing them that in our experiment we gather anonymous data, and we ask for the students’ NABC IDs (IDs used at the National Assessment of Basic Competences), which enables us to link our experimental data to anonymous NABC data on school performance and socioeconomic background at the individual level. We also informed the education providers that we would collect these IDs. The NABC ID is a hash-code of the students' educational IDs that is used solely to identify students inside the NABC surveys and is not connected to any other datasets. Students could only begin the experimental games after typing their IDs, and no other personal information was requested. Only two pupils chose not to participate in our experiments.

The sessions were carried out during school hours since we went to the schools and carried out the experiment there. This meant two important things: we had to adjust to the time schedule of the schools, and participants in a particular session were always classmates. Because of the time adjustment needs, each session lasted about as long as a normal lecture in a Hungarian high school (usually 45 min, followed by a 15 min break). As a result, we only had 45 min (at most 60 min) to conduct the experiment with a class.

Participants being classmates in all sessions was an essential aspect of our experiment since it enabled us to assess in-group and out-group favoritism as well as other class-level traits. Some of the tasks were individual tasks, with payoffs unrelated to the decisions of other participants. Other parts of the sessions required strategic interaction, thus the payoffs were impacted by the decisions of two classmates. The software we used to design the experiment (z-Tree [5]) generated student pairs at random in these instances. Pairing took place at the end of each session, after we had gathered information regarding each student's decision in every situation. When there were an odd number of participants in the room, one "pair" of pupils was a group of three. In these instances, the payoff of one student was impacted by only one of the other two students in the group of three. This was likewise randomly decided by our software.

Participants used school computers in just two Budapest schools, in all other occasions we brought our laptops and turned one classroom in each school into our lab for one day. Groups of students then took turns by the hour, completing a session in one lecture's time. Because schools usually do not have or only have tiny computer rooms, it was simpler to bring our devices with the required software and settings.

When participants entered the lab, they were allowed to choose a seat. After everyone was seated, an experimenter read aloud the instructions which students could also follow on a sheet of paper in front of them. Then, all questions – if there were any – were answered. On the experiment's start screen, a short version of the instructions was displayed again along with a short text reassuring participants that their answers would remain anonymous.

There were no time limits in the various tasks (with the exception of the real-effort task to assess competitiveness, see later), so participants could proceed in their own pace. The only limitation, as previously stated, was that we had to complete each session before the next lecture started. We also informed participants that there might be some variation in how long it would take to different individuals to decide in different tasks, and we urged them to be patient even if they finished early. In reality, there was a big variation in the time spent on the experiment by participants, but there were no incidents as a result of having to wait for those who required more time to decide.

Fortunately, there were no incidents involving misbehavior throughout the sessions either. On each occasion, at least two experimenters were present in the room to ensure that everything went smoothly. We informed participants in the instructions that we did not allow misbehavior (speaking to others, looking at others’ screens, etc.) and that such behavior might result in the offending participant being expelled without compensation.

To incentivize decision making in our experiment, we handed out meal vouchers for the school cafeterias. It was made clear to the students prior to each session that they would make choices in 8 tasks, but at the end of the session, the computer would choose only one of the tasks at random for payment (same for everyone in the classroom). We also explained that many of the tasks required multiple decisions, and if one of these tasks are chosen for payment, only one of the decisions (also determined by the computer randomly) would be paid out. Because we paid students in hundred-forint vouchers, all payoffs were rounded to the nearest hundred HUFs, and the expected payoff was approximately 1000 HUF (around 3 EUR), the typical price of a full meal at a school cafeteria. We paid no show-up fee for students as we conducted our experiment during their lecture time at their schools.

Participants were told about all these payout specifics right before each session (in the instructions), and they were paid after everyone in their school group had completed all tasks. If one of the two time-preference tasks (in which students had to choose between various amounts of money to be received at different points of time) was chosen for payment, everyone was paid according to their choice regarding the amount of money and the date of payment. The amounts requested at the time of the experiment were distributed immediately at the end of each session. Students, however, who wanted to have a specific amount two, four, or six weeks later had to deposit their vouchers in an envelope, which we placed at the school secretariat, requesting the school's administration to distribute them two, four, or six weeks later (as indicated on each envelope).

It has not been clear in which order our 8 tasks should follow each other. When determining this, we took several factors into account. First, we wanted to separate the two time preference tasks because participants could have inadvertently tried to be consistent by making the same choices if these two tasks had been adjacent. Since the two dictator games included the same choice but with distinct reference groups, we left these questions close to each other. The only activity that might have had a greater impact on the participants' emotions was the competitiveness task, since participants were put in a competitive environment that some of them may not have enjoyed. Furthermore, feedback on their performance was given after each competition round (see details later). We shifted the competitiveness task to the very end of the experiment to prevent having the experience in this game influence students’ decisions in other tasks. We did not provide feedback to participants after any of the other tasks in order to prevent the possibility that the result of one task might influence their decision in the next tasks.

We gave detailed feedback to the schools after each visit. In the feedback, we described briefly what preferences the various tasks assessed, and we provided the key descriptive statistics, comparing them to the main results of the literature. We also briefly compared how different school groups performed.

### 2.2 Time preferences (task 1 and 6)

Time preferences have at least two relevant aspects: patience (how an individual values the future relative to the present), and time consistency (which indicates if this relative valuation is the same at different points in time). To capture both aspects of time preferences, we needed two different time horizons.

In task 1, subjects had to select between getting a lesser amount now or a greater amount in 2 weeks, and in task 6, they had the same choice, but the dates were 4 weeks vs. 6 weeks. Participants had to make 5 interdependent decisions on both horizons, following the staircase (or unfolding brackets) method [1,6]. This technique makes effective use of a limited number of questions to identify the point of indifference between the earlier and later payoffs. In each instance, the earlier amount was fixed (1000 HUF), while the later amount (X) was adjusted adaptively based on the previous choices. For example, if a participant selects 1000 HUF now rather than X = 1540 HUF in two weeks, we conclude that her indifference point is greater than 1540 HUF (as 540 HUF is not enough for her to wait for two weeks), therefore X is increased in the next question. Similarly, selecting the later amount indicates a reduction in X in the following question. The value of X ranged from 1030 to 2150 HUF.

Five questions provide a fair estimate of the indifference point, allowing us to determine how much we have to pay in order for the participant to be willing to wait two weeks for payment. Assume that in the final question of task 1 (payment now vs. in 2 weeks), a participant decides to get 1730 HUF in 2 weeks instead of 1000 HUF today. Then (as a result of the reward structure), we know that her indifference point is between 1730 HUF and the nearest lower amount (which is 1650 HUF). For practical reasons, we assume that her indifference point is 1650 in this instance, and thus she requires 650 HUF compensation for waiting two weeks for the payment. If the same person has the same indifference point in task 6 (4 weeks vs. 6 weeks), she is time consistent, while a lower indifference point in task 6 indicates that she is present-biased. Time consistent individuals trade off earlier and later benefits in the same way at different points in time. In contrast, present-biased (future-biased) individuals are less (more) patient in the near future than later on.

The supplementary file “Figure1.png” shows the tree of the first 5 choices that we used during the experiment to measure time preferences. All numbers in nodes represent a certain amount of money that could be offered to participants as an alternative sum received later instead of 1000 HUF received earlier. Blue lines represent the routes to the next, higher offer if the sum in the starting node was declined by the participant (and the earlier 1000 HUF was accepted). Red lines represent the routes to the next, lower amount offered, if the participant accepted the sum in the starting node instead of the 1000 HUF.

In the 6th choice in both tasks (which was a control question), the later amount either was very high (3000 HUF, which is triple the amount of the immediate payment) or lower than 1000 HUF (900 HUF). In cases where the participant always chose the earlier 1000 HUF instead of the later but larger amounts (including choosing 1000 HUF earlier instead of 3000 HUF 2 weeks later), the indifference amount variable is set to missing, as these choices imply an extraordinarily high and not calculatable discounting of the future. Always choosing the later (and, in the first five choices, the higher but decreasing) amount and then choosing a later 900 HUF instead of an earlier 1000 HUF indicates negative discounting, and the indifference amounts are set to missing in these cases too.

We explained to participants that if one of these tasks was selected for payment, the computer would choose one of the first five choices at random and their choice in that decision would determine how much they earn. For example, if a student selected 1540 HUF in two weeks instead of 1000 HUF today, she would get the 1540 HUF in two weeks from the school administration, as we previously stated.

### 2.3 Risk preferences (task 4)

Risk preferences describe how a person handles a decision with an uncertain result. Crosetto and Filippin's [2] bomb risk elicitation task was utilized. The following short story was described to participants in this task. There are 100 numbered boxes in a room, with one box containing a bomb. The bomb has an equal probability of being in any of the boxes. Participants must choose the number of boxes they want to take out of the room. The boxes, however, may only be gathered in the order in which they are numbered. Earnings grow as the number of boxes gathered that do not contain the bomb rises, but if the bomb is in one of those boxes, the participant earns nothing. Participants' risk preferences are proxied by how many boxes they are willing to take out.

We made participants aware that if this task was chosen to be paid out, zTree would choose a random number between 1 and 100 to indicate the location of the explosive. Everyone who took out a lower amount of boxes than that number would earn 20 HUF per box collected, but those who also took the bomb out would not earn anything.

Choosing 100 boxes is equivalent to a sure explosion and zero earnings. We set the risk-taking measure to missing if the student took 100 boxes.

### 2.4 Social preferences

#### 2.4.1 Altruism/generosity (task 2 and 3)

The dictator game was used to assess altruism (or generosity). We set up two dictator games with different social distance between the dictator and the recipient. In the first (task 2), each participant had 2000 HUF which they could divide between themselves and a classmate (that is, a participant in the same session). The proportion of the original 2000 HUF that is donated to the other student shows the degree of generosity which we report in a percentage form. It was made clear to students that if this task was payoff-relevant at the end of the session, we would form student pairs at random, and the computer would randomly choose one of the participants in each pair whose choice would be implemented. In task 3, the same decision had to be made but this time the recipient was a random schoolmate rather than someone from the room. This was a fictitious task, as it could not have been implemented.

#### 2.4.2 Cooperativeness (task 5)

The public goods game was employed in task 5 to assess cooperativeness. We used a two-person version instead of creating groups of four as is typical in most public goods game studies. That is, we matched each student with another participant from the same session randomly. They were each endowed with 1000 HUF and had to decide how much of that endowment to offer to a common project without knowing how much the other participant had contributed. The part of the 1000 HUF which they decided to keep became part of their potential payoff. The marginal per capita return was 75%, which means that each participant got 75% of the total contribution regardless of how much they contributed individually. Our measure of cooperativeness is the percentage of the initial 1000 HUF that the individual contributed to the common project.

Participants could test different scenarios before making a decision about their contribution. They were presented with two sliders on the decision screen ranging from 0 to 1000, the first representing their contribution and the second representing their partners’. They could see the reward implications of various contribution combinations by adjusting the sliders.

#### 2.4.3 Trust (task 7)

We used the trust game, or commonly known as the investment game by Berg et al. [7], to assess trust and trustworthiness. The game was divided into two stages. In stage 1, each participant chose how much of their initial 1000 HUF endowment to send to a randomly selected recipient in the room, knowing that the money sent would triple at the receiver, and in stage 2, the receiver may give any percentage of that greater amount back. The proportion of 1000 HUF sent had to be rounded to 100 HUFs, and that is our measure of trust (also reported in percentage form, as a share of the 1000 HUF). In stage 2, everyone took on the position of the receiver, and they had to decide how much of the 3*X of all hypothetical X amounts received (*X* = 0, 100, 200,...1000) they would return to the sender. Thus, we have answers to all scenarios, which makes it possible to assess participants' trustworthiness. More specifically, we compute the percentage of the money returned for each conceivable amount received and tripled, and take the average of these shares. This average percentage of money sent back is the measure of trustworthiness. Everyone made a decisions as both senders and receivers.

We explained to participants that in case this game was paid out later, the computer would create random pairs out of students in the room, with one player in each pair being randomly selected as the sender and the other as the receiver, and their associated decisions would determine the payment.

### 2.5 Competitiveness (task 8)

In the last task, we utilized the game design of Niederle and Vesterlund [3] to assess how competitive students are. We only changed the real-effort task. Participants had to count zeros and ones in 5 × 5 matrices (see in [8]) instead of adding up integers. The aim was to solve as many matrices in 60 s as they could. There were three rounds all of them lasting for 60 s.

The game began with a piece-rate round where players were paid 100 HUF for each successfully completed matrix. In round 2, a tournament-style payment scheme was used, with only the top 25% of competitors earning money. Students in the top quarter earned 4 times as much per matrix solved as in round 1. In round 3, participants had to choose between the piece-rate or the tournament-based payment schemes, and this decision indicates their competitiveness (with the ones opting for the tournament scheme being competitive). At the end of round 3, we asked participants how they evaluated their performance in round 1 (piece-rate) and round 2 (tournament). They had to guess whether they were in the first/second/third/fourth quartile based on their performance, the first being the best. Those who guessed right got 300 HUF extra (if this task was chosen for payment).

If the computer selected this task for payment, it also chose one of the rounds at random, and participants were paid based on their performance in that stage.

### 2.6 Linking and cleaning NABC data

The following variables were calculated using data from the NABC: employment status of the father, educational attainment of parents, child support received by the student's family, number of books at home, standardized mathematics and literacy test scores, GPA, grades, age, gender. The first four variables (originally categorical variables) were turned into dummy variables, with missing as a separate category. In case of the GPA and the grades, missing values were imputed with the sample mean, but we also created a separate dummy indicating imputed (and initially missing) values. Test scores were z-standardized to mean 0 and standard deviation 1. The NABC hash codes were dropped.

## Ethics Statement

Informed consent was obtained for experimentation from the participants and their families. Two students opted out.

The experiments were run in Hungarian, and the related legal documents are available in Hungarian here: https://kti.krtk.hu/kapcsolat/altalanos-tajekoztato-a-kiserletekrol/.

## CRediT authorship contribution statement

**Dániel Horn:** Conceptualization, Methodology, Validation, Investigation, Writing – review & editing, Supervision, Funding acquisition. **Hubert János Kiss:** Conceptualization, Methodology, Validation, Investigation, Writing – review & editing. **Tünde Lénárd:** Software, Investigation, Data curation, Writing – original draft.

## Declaration of Competing Interest

The authors declare that they have no known competing financial interests or personal relationships which have or could be perceived to have influenced the work reported in this article.

## Data Availability

Preferences of adolescents – A dataset containing linked experimental task measures and register data (Original data) (Mendeley Data).

## Footnotes

1 There was a pilot school in our sample at the very beginning of the experiment where we conducted sessions in 2018. As several tasks were altered after running the pilot and the measures taken there are not comparable to the data from the other schools, we do not include the pilot school in our published dataset.

2 Class in the Hungarian context refers to a smaller group of students (usually around 30 students) from the same school-grade who visit most of their lectures together and have a common head teacher.
